# An investigation into the causes of race bias in artificial intelligence–based cine cardiac magnetic resonance segmentation

**DOI:** 10.1093/ehjdh/ztaf008

**Published:** 2025-02-24

**Authors:** Tiarna Lee, Esther Puyol-Antón, Bram Ruijsink, Sebastien Roujol, Theodore Barfoot, Shaheim Ogbomo-Harmitt, Miaojing Shi, Andrew King

**Affiliations:** School of Biomedical Engineering & Imaging Sciences, King's College London, UK; School of Biomedical Engineering & Imaging Sciences, King's College London, UK; HeartFlow Inc, London, UK; Guy's and St Thomas' Hospital, London, UK; School of Biomedical Engineering & Imaging Sciences, King's College London, UK; School of Biomedical Engineering & Imaging Sciences, King's College London, UK; School of Biomedical Engineering & Imaging Sciences, King's College London, UK; College of Electronic and Information Engineering, Tongji University, China; School of Biomedical Engineering & Imaging Sciences, King's College London, UK

**Keywords:** Cardiac magnetic resonance, Artificial intelligence, Cardiac segmentation, Cardiac classification, Bias

## Abstract

**Aims:**

Artificial intelligence (AI) methods are being used increasingly for the automated segmentation of cine cardiac magnetic resonance (CMR) imaging. However, these methods have been shown to be subject to race bias; i.e. they exhibit different levels of performance for different races depending on the (im)balance of the data used to train the AI model. In this paper, we investigate the source of this bias, seeking to understand its root cause(s).

**Methods and results:**

We trained AI models to perform race classification on cine CMR images and/or segmentations from White and Black subjects from the UK Biobank and found that the classification accuracy for images was higher than for segmentations. Interpretability methods showed that the models were primarily looking at non-heart regions. Cropping images tightly around the heart caused classification accuracy to drop to almost chance level. Visualizing the latent space of AI segmentation models showed that race information was encoded in the models. Training segmentation models using cropped images reduced but did not eliminate the bias. A number of possible confounders for the bias in segmentation model performance were identified for Black subjects but none for White subjects.

**Conclusion:**

Distributional differences between annotated CMR data of White and Black races, which can lead to bias in trained AI segmentation models, are predominantly image-based, not segmentation-based. Most of the differences occur in areas outside the heart, such as subcutaneous fat. These findings will be important for researchers investigating performance of AI models on different races.

## Graphical Abstract

**Graphical Abstract:**
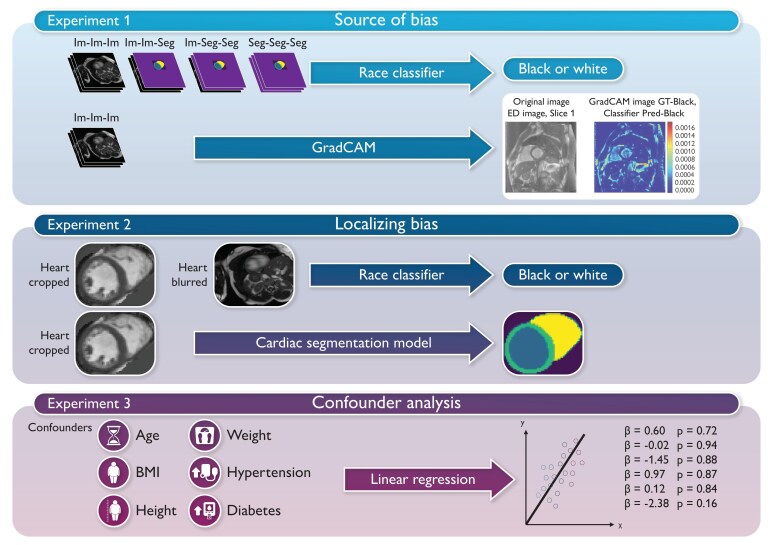

## Introduction

Cardiac magnetic resonance (CMR) imaging is widely used to acquire images for diagnosis and prognosis of cardiovascular conditions. Artificial intelligence (AI) methods are increasingly being used to automate the estimation of functional biomarkers from cine CMR by automatic delineation (segmentation) of cardiac structures.^1–3^ However, recent work has shown that AI CMR segmentation models can exhibit different levels of performance for different protected groups, such as those based on race^4–6^ or sex^7^ (i.e. they can be *biased*).

Bias in AI models is often the result of a distributional shift between the data of subjects in different protected groups. Combined with imbalance in the training data, these distributional shifts can lead to the internal representations of AI models being optimized for the protected group(s) who were in the majority in the training data.^6,8^ This can lead to underrepresented groups having lower performance, for example, poorer quality AI-produced segmentations. This is problematic as the lower performance can translate to greater errors when AI is used in the estimation of patient health and consequently worse clinical outcomes, exacerbating existing health disparities. Note that the ability of AI to recognize patient race which has recently been reported across a range of medical imaging modalities^9^ is a symptom of the distributional shifts which can lead to bias. Artificial intelligence–recognized patient race could be misused and therefore also represents a potential danger to patients. However, in this paper, we focus on the problem of AI (segmentation) model bias, not the potential misuse of AI-recognized patient race.

To properly address the bias in AI segmentation models, it is important to understand its causes, but these are not yet well understood. This paper presents an investigation into the causes of race bias in AI-based CMR segmentation.

## Methods

### Dataset

The dataset used in the experiments described in this paper comprised cine short axis (SAX) CMR images from 436 subjects from the UK Biobank.^10^ The images were acquired in Cheadle, UK, on a clinical wide bore 1.5 Tesla scanner (MAGNETOM Aera, Syngo Platform VD13A, Siemens Healthcare, Erlangen, Germany). A balanced steady-state free precession sequence was used with TR/TE = 2.6/1.1 ms and flip angle 80°. The slice thickness was 8.0 mm with a slice gap of 2 mm. Each voxel was 1.8 mm × 1.8 mm, giving a resolution of 1.8 × 1.8 × 8.0 mm. For each subject, typically 7–13 SAX slices were available at 50 time frames covering the cardiac cycle. The demographic information of the subjects can be found in *Table 1*.

**Table 1:** Clinical characteristics of subjects used in the study (R1.4)

Health measure	Overall	White	Black
# of subjects	436	218	218
Age (years)	58.9 (7.0)	58.9 (7.0)	58.8 (6.9)
Weight (kg)	80.6 (16.6)	79.3 (17.0)	82.0 (16.1)
Standing height (cm)	170.4 (9.22)	171.3 (9.1)	169.4 (9.2)
Body mass index	27.7 (4.9)	26.9 (4.6)*	28.6 (5.1)*

Mean values are presented for each characteristic with standard deviations given in brackets. Statistically significant differences between subject groups and the overall average are indicated with an asterisk * (*P* < 0.05) and were determined using a two-tailed Student’s *t*-test.

**Figure 1:**
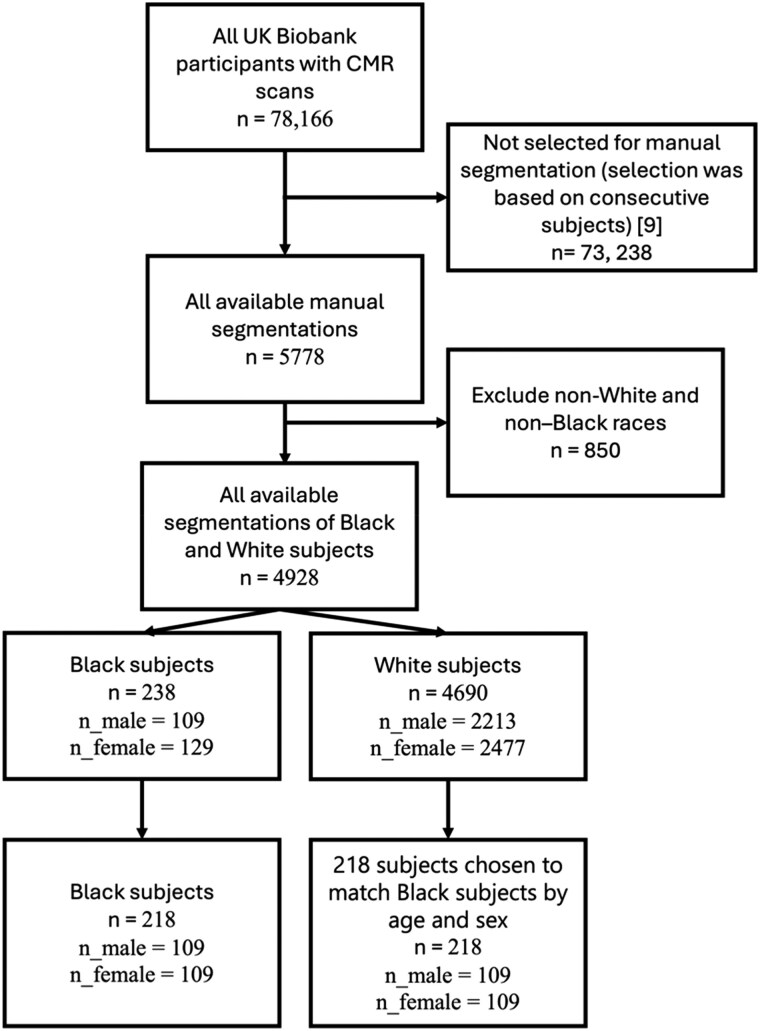
Case selection flowchart showing the inclusion/exclusion criteria for subjects in the study. All available manual segmentations for the study were used, non-White and non-Black subjects were excluded, and then, the subjects were split into Black and White groups. There were 109 Black males, so 109 Black females, White females, and White males were chosen to match this number to create a balanced study (R1.6).

The subjects selected for potential inclusion from the full UK Biobank CMR cohort were those with available manual ground truth segmentations (4928 out of 78 166 subjects, 4690 White subjects and 238 Black subjects).^11^ Other selection/exclusion criteria are depicted in *Figure 1*. Note that disease and risk factors were not considered as exclusion criteria so the cohort included subjects who had diabetes mellitus, hypertension, current or ex-smokers, vascular problems diagnosed by a doctor, cancer, and those taking medication for cholesterol, blood pressure, or diabetes. The manual segmentations were of the left ventricular blood pool (LVBP), left ventricular myocardium (LVM), and right ventricular blood pool (RVBP) and were performed for the end-diastole (ED) and end-systole (ES) images. Therefore, only the ED and ES frames were used in our experiments. Manual segmentation was performed by outlining the LV endocardial and epicardial borders and the RV endocardial border using cvi42 (version 5.1.1, Circle Cardiovascular Imaging Inc., Calgary, Alberta, Canada).^11^ A panel of 10 experts was provided with the same guidelines and one expert annotated each image. The selection of images for annotation included subjects with different sexes and races, and images were randomized between the experts. The experts were not provided with demographic information about the subjects. Any problematic cases were flagged and resolved by the consensus of at least three members of the team with relevant knowledge. Segmentation consistency was tested by assessing the inter- and intra-observer variability of atrial and ventricular measurement using a random selection of the CMR examinations.

From the available data, a cohort of 218 Black subjects out of a pool of 238 available Black subjects was selected for use in all experiments. This cohort was chosen to have 109 males (all available Black males) and 109 females to minimize the impact of possible sex bias. To select the White subjects, a matched pairs design was used, in which White subjects with matching age (±1 year) and sex to each Black subject were chosen at random from the available pool of 4690 White subjects with ground truth segmentations. For each subject, the ED and ES frames of all SAX CMR slices and their corresponding ground truth segmentations were utilized in the experiments. Demographic and health data from the subjects were acquired from the UK Biobank database including the subjects’ age, standing height, weight, body mass index (BMI), resting heart rate, systolic and diastolic blood pressure, left ventricular stroke volume (LVSV), left ventricular ejection fraction (LVEF), left ventricular end-diastolic mass (LVEDM), HDL cholesterol, cholesterol, diabetes status, hypertension status, hypercholesterolaemia status, smoking status, and date of the MRI scan.

### Models used

To perform the investigations into the source of bias, we employ two types of AI model: a classification model and a segmentation model.

ResNet-18 is a deep convolutional neural network (CNN) for classification consisting of 18 layers.^12,13^ The network has residual blocks and skip connections which can be used to form deep networks. For the classification experiments (Experiments 1 and 2 in the Results), the model was trained for 100 epochs with an initial learning rate of 0.001 which decreased by a factor of 10 every 50 epochs. The loss function used was binary cross entropy, and the model was optimized using stochastic gradient descent. The batch size was 16. The images were augmented using random mirroring, rotating, scaling, and translation. As the images are greyscale, no colour intensity transformations were used. Each model was trained 10 times with different random seeds and train/validation splits, and the mean and standard deviation for these 10 runs are reported.

For the classification network, we also employed the gradient-weighted class activation mapping method, or GradCAM, which is a visualization and interpretability method.^14^ The gradients of the target class (in our case, race) in the last convolutional layer of the classification network were visualized to produce a heatmap which shows the areas of an image that were most important for the classification decision.

For the segmentation experiments, we used nnU-Net, a self-adapting framework for segmentation of biomedical images.^14^ The network automatically adapts to the imaging modality and changes training parameters such as the patch size, batch size, and image resampling. The nnU-Net v1 model consists of an encoder and decoder structure which form a ‘U’ shape,^14^ allowing the network to learn a more abstract representation of the images. For the segmentation experiments (Experiments 1 and 2 in the Results), the model was trained for 500 epochs with an initial learning rate of 0.01. The loss function used was a combined Dice and cross entropy loss. The model was optimized using stochastic gradient descent with a ‘poly’ learning rate schedule, where the initial learning rate was 0.01 and the Nesterov momentum was 0.99. A batch size of 16 was used. During training, data augmentation was applied to the images including mirroring, rotation, and scaling. Cross-validation was performed on the training set, resulting in five models, which were used as an ensemble for inference on the test set.

### Statistical evaluation

Classification accuracy was evaluated using overall accuracy, sensitivity, and specificity. Differences in performances were evaluated using a two-tailed Student’s *t*-test of the accuracies of the 10 runs. Segmentation performance was evaluated using the Dice similarity coefficient (DSC) which measures the overlap between ground truth and predicted segmentations where 1 is a perfect overlap and 0 is no overlap. Confounder analysis was performed using linear regression models in SPSS Statistics (IBM Corp. Released 2023. IBM SPSS Statistics for Macintosh, Version 29.0.2.0 Armonk, NY: IBM Corp).

## Results

The experiments performed using the data and models described above aimed to investigate three aspects of the bias in AI CMR segmentation performance as detailed below.

### Experiment 1: source of bias

As stated earlier, bias in AI models can arise from distributional shifts between the data of different protected groups. However, the distributional shift can be in the images, the ground truth segmentations, or a combination of both. Understanding the origin of the bias in trained segmentation models is important when deciding on strategies to address it. Therefore, the first experiment aimed to assess the extent of the distributional shift between the CMR images and/or the ground truth segmentations. The findings of this experiment are used to inform and motivate subsequent experiments aimed at more closely identifying the potential source of bias.

To quantify the extent of the distributional shifts, we trained ResNet-18 models to classify the race of the subject (White vs. Black) from a single SAX CMR image and/or segmentation. The SAX CMR images and ground truth segmentations of the 218 Black and 218 White subjects were randomly split at the subject level into training and test datasets with 352 and 84 subjects respectively (ensuring that both images from each matched pair were in the same split).

**Figure 2:**
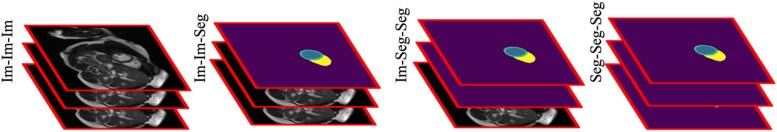
An illustration of the combination of images and segmentations used as input to the protected attribute classifiers. Im, original cardiac magnetic resonance image; Seg, ground truth segmentation (R1.7).

The classifier was trained with three channels as input. Note that the images are not RGB images, but we used the three channels to incorporate image and/or segmentation information into the models. Therefore, each of the three channels was either a greyscale CMR SAX image or a corresponding greyscale segmentation. To assess the relative distributional shifts between images and ground truth segmentations, we used four different combinations of images (Im) and segmentations (Seg): Im-Im-Im, Im-Im-Seg, Im-Seg-Seg, and Seg-Seg-Seg, as illustrated in *Figure 2*.

*Table 2* shows the results for classifying Black and White race subjects. The highest accuracies were achieved when images were used, either on their own or in combination with segmentations. The accuracy of the Seg-Seg-Seg dataset was the lowest but still higher than random chance. Using a two-tailed Student’s *t*-test between the accuracies of the 10 runs for each of the datasets, the only significant differences were between the Seg-Seg-Seg dataset and the other three datasets which contained images (*P* < 0.0001 for all). We also plotted a histogram of the mean image intensities for White and Black subjects (see Supplementary material online, *Figure S1*) which revealed a slight distributional shift but not enough to explain the race classification accuracy.

**Table 2:** Accuracy for experiment on classifying the subjects by race (Black and White)

Image type	Accuracy	Sensitivity	Specificity
Im-Im-Im	**0.959** (**0.004)**	**0.966** (**0.013)**	0.951 (0.014)
Im-Im-Seg	0.957 (0.010)	0.959 (0.011)	**0.956** (**0.018)**
Im-Seg-Seg	0.955 (0.007)	0.961 (0.013)	0.948 (0.010)
Seg-Seg-Seg	0.742 (0.005)	0.727 (0.011)	0.765 (0.020)

The results show the mean (standard deviation) over 10 repeat runs. The highest result for each measure is shown in bold. There were 88 Black and 88 White subjects in the training dataset and 42 Black and 42 White subjects in the test dataset.

The conclusion of this first experiment is that the majority of the distributional shift between races lies in the images rather than the ground truth segmentations.

**Figure 3:**
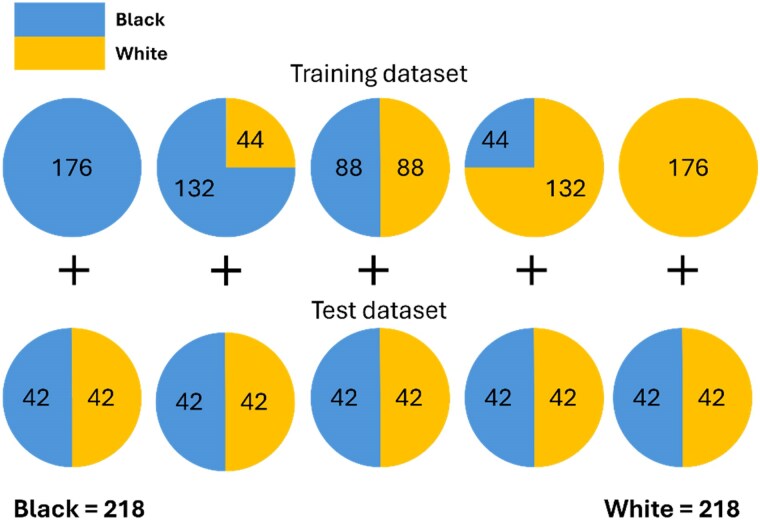
Illustration of the number of subjects used in training and test datasets in Experiment 1.

The next experiment aimed to assess whether, and the degree to which, this distributional shift is also encoded in trained segmentation models. This encoding may suggest that biased representations of the images are being used to produce segmentations, which may contribute to the segmentation bias observed in Lee *et al*.^6^ To answer these questions, we used an approach similar to that described in Glocker *et al*.^15^ First, nnU-Net models were trained to segment CMR images using training data with varying levels of race imbalance. Next, the decoder part of the trained networks was removed. The test set was then fed through the encoder part of the network to produce a latent vector for each test subject. We then used principal component analysis (PCA) to reduce the dimensionality of these latent representations and visualized the results. Furthermore, we investigated whether race could be separated in the reduced dimensional space using logistic regression classification. *Figure 3* illustrates how 176 subjects of each race (Black and White) were used to form five different training sets with different levels of imbalance for this experiment. At the extremes, i.e. 100% Black subjects or 100% White subjects, 176 Black or 176 White subjects were used for training. The other training sets were formed from random subsets of the same 176 White and 176 Black subjects as shown. The 84 subjects in the test set (42 of each race) were constant across all models evaluated. Therefore, in total, 176 + 42 = 218 subjects of each race were utilized for the experiments although not all of them were used in all training sets.

The quantitative results for this experiment are shown in *Table 3*. The PCA-reduced dimensional representations of the images could be classified with a high accuracy of approximately 95% for all models. Visual illustrations of the classification of protected attributes can be found in Supplementary material online, *Figure S2*.

**Table 3:** Accuracy of a logistic regression model classifying the principal components analysis representations of the test cardiac magnetic resonance images fed through the segmentation model encoder

Training dataset split	Black vs. White
100%/0%	0.950
75%/25%	0.950
50%/50%	0.949
25%/75%	0.951
0%/100%	0.949

The conclusion from this experiment is that race appears to be encoded in the latent representations of the trained segmentation models. This suggests that the (predominantly image-based) distributional shift is leading to different encodings for different races, which could be a likely cause of the bias in segmentation model performance.

### Experiment 2: localization of source of bias

The first set of experiments resulted in high accuracy for race classification, suggesting a strong distributional shift. They also suggested that the source of the bias was mainly in the images and that it was being encoded into the segmentation model. Therefore, we next sought to understand which parts of the images were leading to the distributional shift and hence the bias. This is important as knowing about the spatial dependence of the distributional shift could potentially lead to effective mitigation strategies. To visualize the relative importance of the different regions of the image, we used GradCAM^13^ applied to the race classification models.

**Figure 4:**
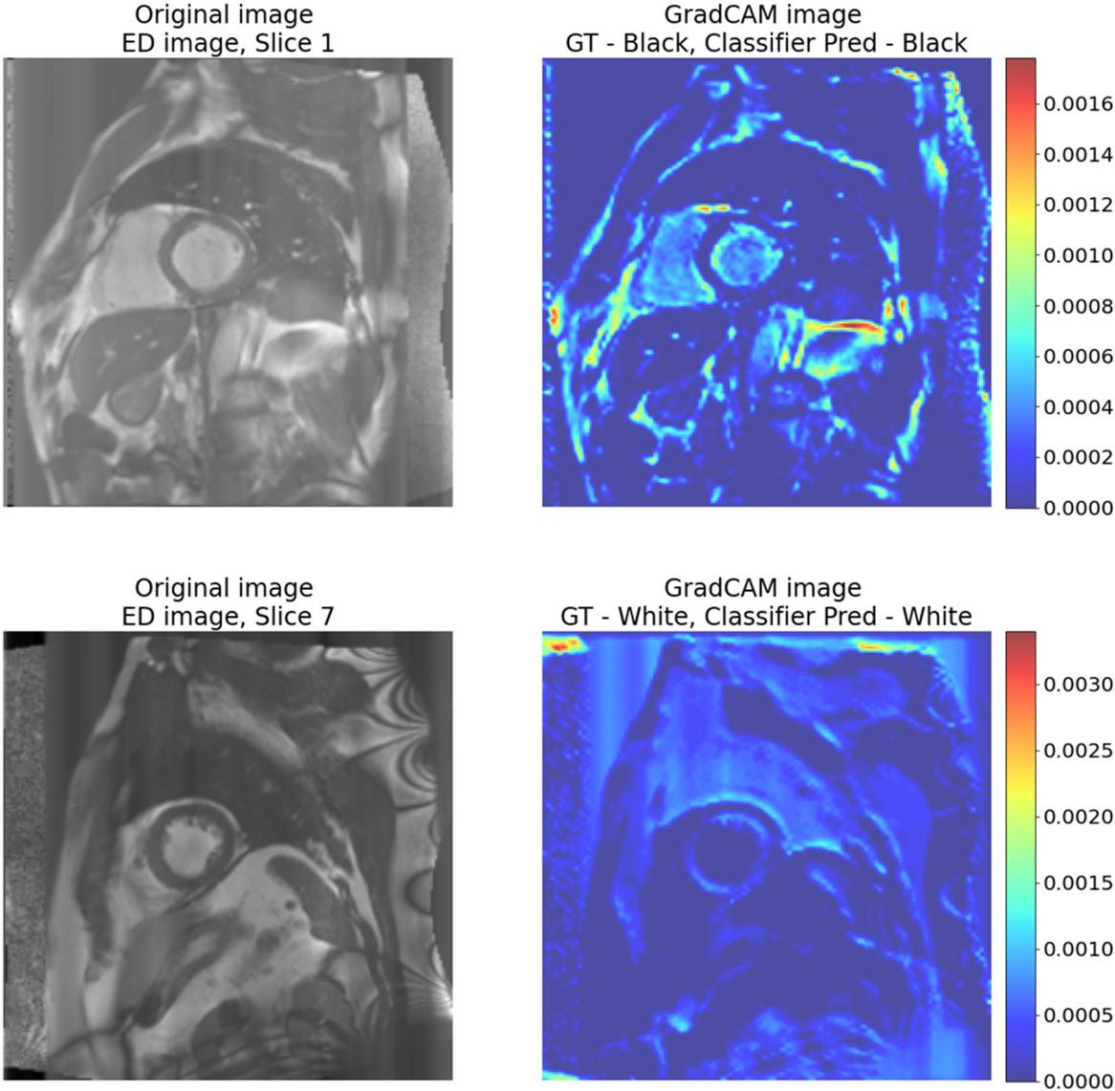
Examples of the normalized cardiac magnetic resonance images and gradient-weighted class activation mapping images for the classification model trained on the Im-Im-Im dataset for Black vs. White subjects. Higher values correspond to important areas used for race classification; lower values correspond to less important areas. The top image displays a heatmap where the non-heart regions have higher activations, and the bottom image shows a heatmap where artefacts have higher activations. ED, end-diastolic; GT, ground truth (R1.11).

The results are shown in *Figure 4* with normalized CMR images and GradCAM images. These representative examples show that for both the Black and White subjects, the most attention is being given to non-heart regions such as subcutaneous fat. Further examples can be seen in Supplementary material online, *Figures S3* and *S4*.

By visual inspection of all test images, we found that 42% of the images had the highest activations in non-heart anatomical regions of the body whereas only 6% had the highest activation in heart regions. The remaining 52% could be classified as ‘activations due to image artefacts’ (50%) and ‘other’ where there were no clear activations in any particular area (2%). These image artefacts become visible after normalizing the images which occurs before model training. The artefacts can be caused by interactions between the magnetic field and body tissues during MR image acquisition. For example, ‘ghosting artefacts’ can cause the skin and fat layers to appear as echoes at regular intervals in an image.^16^

**Figure 5:**
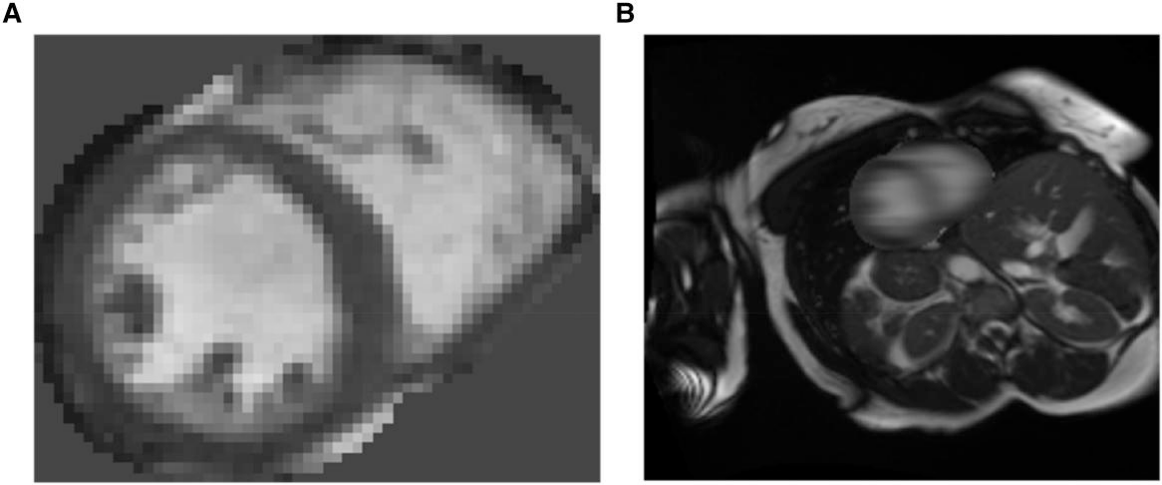
Examples of images including and excluding the heart: (*A*) image cropped around the heart; (*B*) image with the heart blurred.

Based on these results, we next investigated the impact on race classification performance of using different areas of the images as input. We created two further datasets of images: a dataset including only the heart and a dataset excluding the heart. For the first dataset, we cropped the images around the region of the heart using a bounding box based on the ground truth segmentations. All images for a given experiment were cropped to the same size, i.e. the size of the largest heart in the dataset. The images were cropped by finding the contour of the segmentation and calculating the minimum and maximum *x* and *y* coordinates of this contour. The images were then cropped with a bounding box according to these coordinates. For the second dataset, the heart was blurred out using a Gaussian filter, which was applied only at voxels which were labelled as foreground (LVBP, LVM, or RVBP) in the ground truth segmentation. Examples of the images created in this way are shown in *Figure 5*. We then repeated the race classification experiments using these new images and compared with the performance on uncropped images.

As before, the results were averaged over 10 repeat runs with different random training and validation sets and random seeds for training. The results can be seen in *Table 4*. Cropping the images around the heart regions caused the accuracy to decrease by 0.405 to 0.554. Blurring the images only caused the accuracy to decrease by 0.046 to 0.913.

**Table 4:** Classification accuracy for original images, images cropped around the heart, and images with the heart blurred

Image type	Accuracy	Sensitivity	Specificity
Im-Im-Im	**0.959** (**0.004)**	**0.966** (**0.013)**	0.951 (0.014)
Images cropped around the heart	0.554 (0.028)	0.618 (0.061)	0.537 (0.029)
Images with heart blurred	0.913 (0.007)	0.884 (0.010)	**0.952** (**0.014)**

The results show the mean (standard deviation) over 10 repeat runs. The highest result for each measure is shown in bold.

The conclusion of these experiments is that the main source of the distributional shift for races is outside the heart area. This agrees with the GradCAM experiments which showed higher activation in non-heart regions such as subcutaneous fat and image artefacts.

Based on this conclusion, we next investigated the impact of training segmentation models after cropping out the regions of the images which seemed to be leading to the distributional shift between races, i.e. the areas outside the heart. Specifically, the segmentation experiments performed in Lee *et al*.^7^ (using the full CMR images) were repeated using the cropped images. These experiments trained multiple nnU-Net segmentation models using different levels of race imbalance in the training set and evaluated their performance separately for White and Black subjects. The images here were cropped to the same size as the previous classification experiment. The models were trained using the same training parameters as in Lee *et al*.^7^

**Figure 6:**
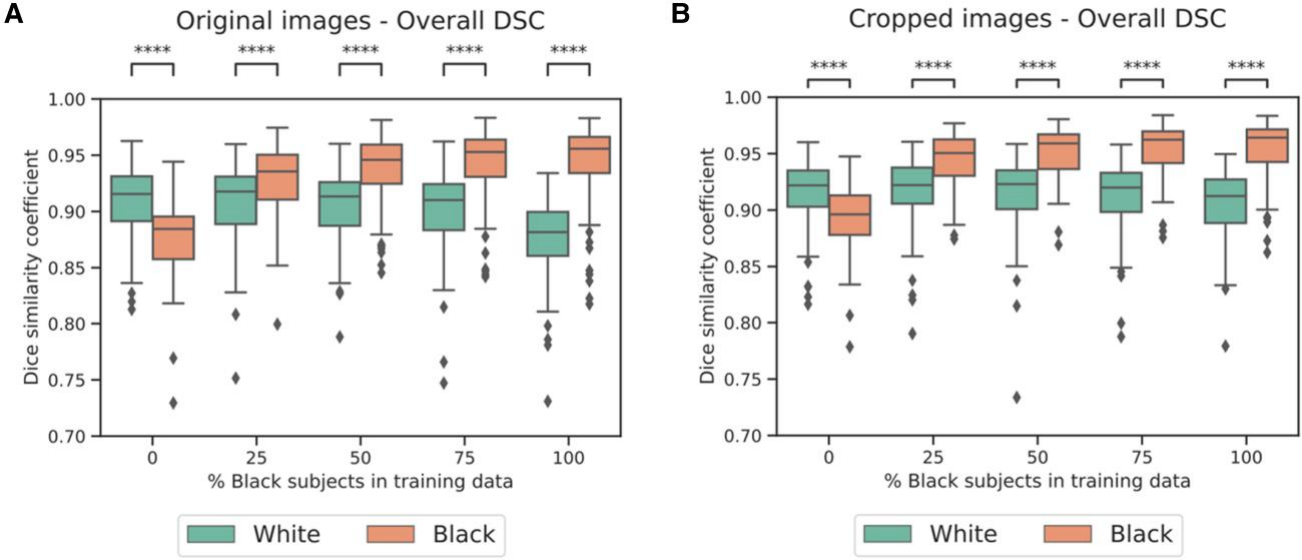
Overall Dice similarity coefficient for segmentation experiments using original (*A*) and cropped (*B*) cardiac magnetic resonance images. Statistical significance was tested using a Mann–Whitney *U* test and is denoted by **** (*P* ≤ 0.0001), *** (0.001 < *P* ≤ 0.0001), ** (0.01 < *P* ≤ 0.001), * (0.01 < *P* ≤ 0.05), and ns (0.05 ≤ *P*).

*Figure 6* shows the results of the segmentation experiments. Comparisons of the predicted end-systolic volumes and ejection fraction for the original and cropped images can be seen in Supplementary material online, *Figure S5*. Compared with using the original images, using the cropped images reduced the range of DSCs for both protected groups at each level of training set imbalance. The differences between DSCs of the two protected groups also reduced although the results remain significantly different. Therefore, cropping the images reduced but did not remove the difference in performance between the Black and White subjects. This is consistent with the classification experiments (*Table 2*) which showed that some distributional shift was present in the heart region.

### Experiment 3: are the biases observed in artificial intelligence cardiac magnetic resonance segmentation due to confounders?

Differences in covariates between protected groups may lead to distributional shifts and consequent bias in the AI CMR segmentation models. These differences may be a cause of the bias in segmentation model performance and understanding these causes can again be helpful in formulating effective mitigation strategies. We investigate whether covariates may be acting as confounders by comparing the DSC values and the covariates of the subjects such as their weight, height, and heart rate. The data are analysed by fitting a linear regression model between covariates and DSC scores.

*Table 5* shows the results of this analysis. The table shows the standardized *β* coefficients and *P*-values for each covariate. The standardized *β* coefficient shows the relative effect of each covariate on the DSC score, with positive coefficients indicating a positive correlation and negative coefficients indicating a negative correlation. The year of the MRI scan was the most predictive of DSC score in the Black subjects. White subjects have no confounders with a *P* < 0.05.

**Table 5:** Parameters from a linear regression model fitting Dice similarity coefficient scores from a segmentation model trained on original, uncropped cardiac magnetic resonance images to covariates for Black and White test subjects

Covariate	Black subjects		White subjects	
	Standardized *β* coefficient	*P*-value	Standardized *β* coefficient	*P*-value
Age	0.060	0.718	− 0.056	0.754
Height	0.126	0.881	− 0.935	0.227
Weight	− 0.240	0.865	2.017	0.206
BMI	− 0.098	0.940	− 1.832	0.172
Heart rate	− 0.241	0.063	− 0.284	0.125
Systolic blood pressure	− 0.253	0.333	− 0.213	0.357
Diastolic blood pressure	0.125	0.604	0.424	0.138
LVSV	− 0.581	0.009	− 0.121	0.713
LVEF	− 0.410	0.012	0.079	0.734
LVEDM	0.453	0.057	0.141	0.688
HDL cholesterol	− 0.321	0.015	− 0.002	0.990
Cholesterol	− 0.015	0.929	− 0.071	0.657
Diabetes	− 0.236	0.155	^a^	
Hypertension	0.032	0.838	− 0.169	0.280
Hypercholesterolaemia	0.175	0.393	0.767	0.446
Smoking	0.1744	0.155	0.041	0.776
MRI year	0.493	< 0.001	− 0.038	0.808

The Dice similarity coefficient scores are from an evenly balanced training dataset.

^a^None of the White subjects had diabetes.

LVSV, left ventricular stroke volume; LVEF, left ventricular ejection fraction; LVEDM, left ventricular end diastolic mass.

## Discussion

In this paper, we have shown that race can be predicted from single SAX CMR images with very high accuracy. However, the accuracy of predicting race from CMR segmentations was noticeably lower, indicating that the distributional shift between White and Black protected groups is mostly in the CMR images as opposed to the manual segmentations.

The GradCAM images showed that the classification networks had the highest activations in non-heart regions such as subcutaneous fat and image artefacts, a result that was further demonstrated by the classification experiments using a dataset with the heart ‘removed’ from the images. The accuracy here remained high whereas we found lower classification accuracy using images cropped tightly around the heart. This suggests that there are fewer race-specific features in the images of the hearts of White and Black subjects and that the distributional shift is mostly in non-heart regions. A similar result was found in Gichoya *et al*.^9^ where occluding regions identified by saliency maps as important for race classification from chest X-ray images caused the accuracy to decrease.

When looking at segmentation tasks, the high classification accuracy of the logistic regression model showed that subjects’ races were encoded in the latent representations of the CMR images, which makes this encoding a likely cause of the bias in segmentation performance. Cropping the images in a similar fashion to the classification experiments reduced, but did not eliminate, the bias found in the segmentation experiments. We speculate that the remaining bias is due to some anatomical differences in the heart region and the fact that it was not possible to completely crop out non-heart regions in all images because of the variability in heart size and the need to maintain a constant image size for AI model training.

The covariate analysis indicated that some variables seemed to be acting as confounders. The LVEDM, LVSV, and LVEF were correlated with DSC score for Black subjects but not for White subjects. Black and White subjects are known to have differences in body composition such as fat distribution and bone density^17^ as well as differences in cardiac anatomy such as Black subjects having higher left ventricular mass.^18^ Although BMI did not show up as a significant confounder, there was a difference in BMI between Black and White subjects (see *Table 1*). Therefore, we repeated the race classification and segmentation experiments using subjects controlled for both age and BMI. We found that the race classification accuracy reduced to 0.554 but that bias in segmentation models remained (see Supplementary material online, *Figure S6*) (R1.5). These distributional shifts may be recognizable to a model and may be used for classification tasks and lead to bias in segmentation tasks. It should be noted that, even though these image features (e.g. subcutaneous fat and image artefacts) are outside of the structures being segmented, AI segmentation models make use of the whole image as contextual information when segmenting cardiac structures. Therefore, differences in non-heart regions like subcutaneous fat and image artefacts can still cause differences between races in the models’ internal representations and hence segmentation model bias.

In our previous work,^5^ we performed a confounder analysis in AI-based CMR segmentation in multiple races including Asian and mixed subjects and found that hypertension was the only covariate that could (partly) explain differences in performance between (multiple) races. In contrast, here we did not find statistical significance for hypertension as a confounder when looking only at Black and White subjects. However, we did find that MRI year was a confounder for Black subjects. We further investigated this and found that, by chance, the White subjects selected in our dataset were on average scanned in earlier years than the Black subjects. It is possible that there were differences in image artefacts over time due to small changes in the acquisition protocol, which would be consistent with the GradCAM activations focusing on artefact areas 50% of the time. Therefore, we reran the segmentation experiments (using the different levels of race imbalance as in Experiment 2) using data which were also matched by MRI year but found no difference in bias characteristics or performance (see Supplementary material online, *Figure S7*). Therefore, we conclude that this was a spurious confounding effect caused by random selection of White subjects who were scanned earlier.

As a recommendation for future development of AI CMR segmentation tools, we suggest that training models using images cropped around the heart may be beneficial. However, this does raise the question of how best to crop images in this way at inference time, when ground truth segmentations are obviously not available. Region-of-interest detection methods such as Mask R-CNN^19^ may be useful for this purpose. It is also likely that cropping on its own will not be enough to remove the bias, and this may need to be combined with other mitigation approaches, such as importance reweighing and over-/under-sampling.^20^ We also emphasize that bias mitigation should not be seen as a substitute for more equal representation in CMR datasets. Our experiments have focused on Black and White subjects, but previous work^6^ has shown that similar bias effects exist for Asian subjects and by sex.^7^ Therefore, we argue for greater representation of all protected groups in CMR datasets.

This work has several limitations. First, all experiments were performed on one dataset (CMR images from the UK Biobank) which contains a specific distribution of subjects. Future work should validate our findings with other datasets which contain subjects of different ethnicities as well as images acquired with different CMR imaging protocols, sequences, and magnetic field strengths. Second, we were only able to use a relatively small number of subjects due to the limited number of available ground truth segmentations from Black subjects. We recommend that further ground truth segmentations should be produced from minority groups, although this can be expensive and labour-intensive. Finally, more confounders could have been investigated in Experiment 3 such as the Townsend deprivation index and postcode.

In conclusion, we have performed a series of experiments to investigate the cause of AI CMR segmentation bias. Our conclusions are as follows: (i) the distributional shift between White and Black subjects is mostly, but not entirely, in the images rather than the segmentations, (ii) differences in body fat composition outside of the heart are a likely cause of the distributional shift and hence the bias, and (iii) cropping the images around the heart reduces but does not eliminate the bias. Our results will likely be valuable to researchers aiming the train fair AI CMR segmentation models in the future.

## Supplementary Material

ztaf008_Supplementary_Data

## Data Availability

The UK Biobank datasets are publicly available for approved research projects. Requests to access these datasets should be directed to https://www.ukbiobank.ac.uk/.
