# Supplementary material for: An investigation into the causes of race bias in artificial intelligence–based cine cardiac magnetic resonance segmentation
Source: Eur Heart J Digit Health. 2025 Feb 24;6(3):350–8. doi: 10.1093/ehjdh/ztaf008 (PMC12088717; doi:10.1093/ehjdh/ztaf008)
Supplement: ztaf008_Supplementary_Data [file ztaf008_supplementary_data.docx]

# Supplementary information

Experiment 1: source of the bias

1. There are distributional differences in the pixel intensities of the images of Black and White subjects. Supplementary Figure 1 shows the distribution of mean image intensities for each image and the mean intensities following normalisation (R1.3).


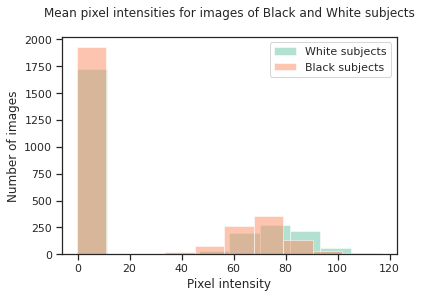


Supplementary Figure 1: Distribution of image intensities for images of Black and White subjects

1. For protected attribute classification, all datasets were trained using a model which was pre-trained on images from the ImageNet1k dataset, apart from the Seg-Seg-Seg dataset which was trained from scratch using randomised weights.
2. A visual representation of the decision boundary used for the classification of latent space representations of images can be seen in Supplementary Figure 2.


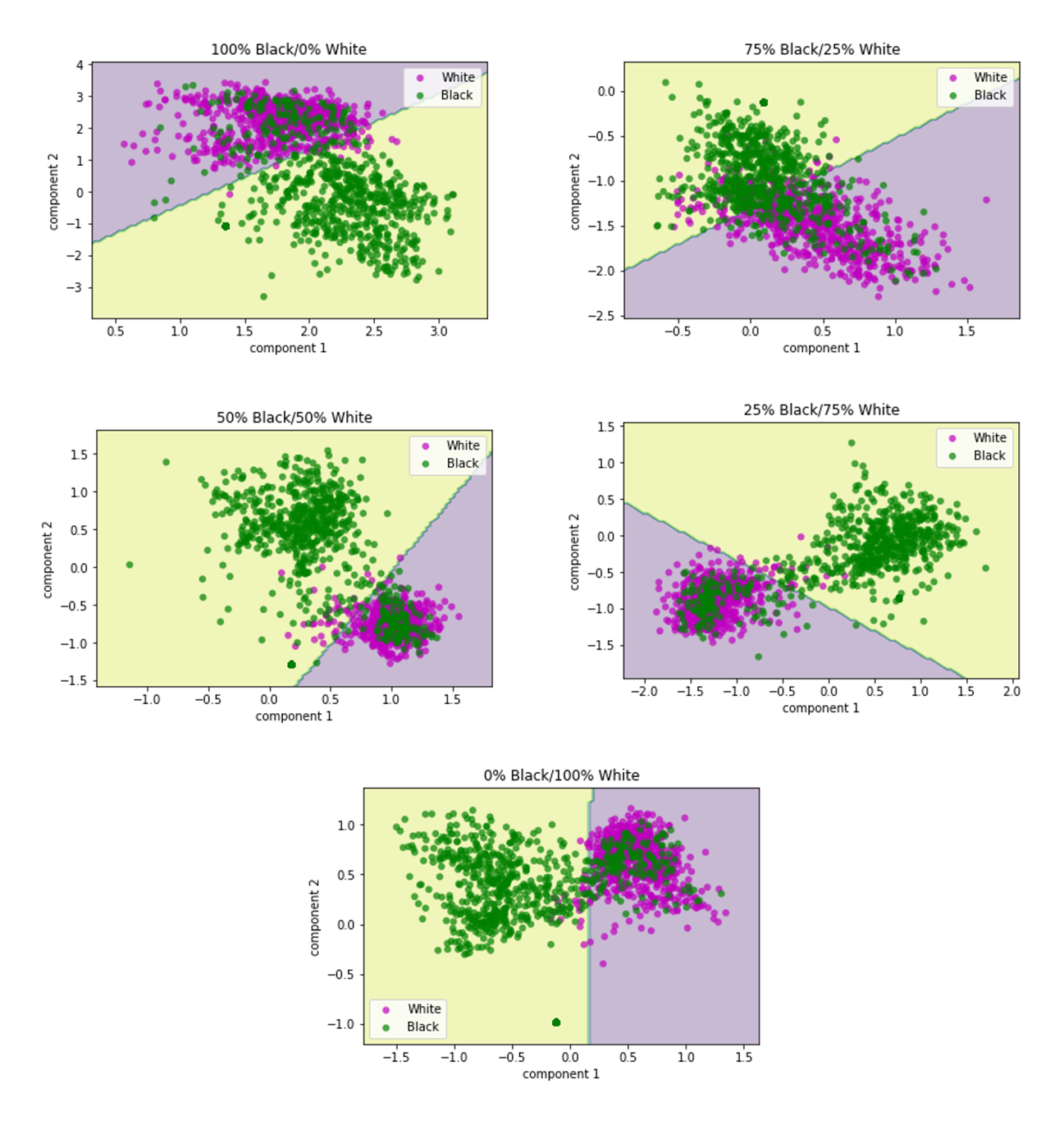


Supplementary Figure 2: Component 1 and 2 of PCA on latent space representations of CMR images from nnU-Net.

Experiment 2: localisation of the source of the bias

1. Before plotting, all GradCAM heatmaps were smoothed using a Gaussian blur with kernel size (3,3) and standard deviation chosen from a uniform distribution between 1 and 2 chosen by visual inspection. Further examples of GradCAM images can be seen in Supplementary Figure 3 and Supplementary Figure 4 using the Im-Im-Im dataset.


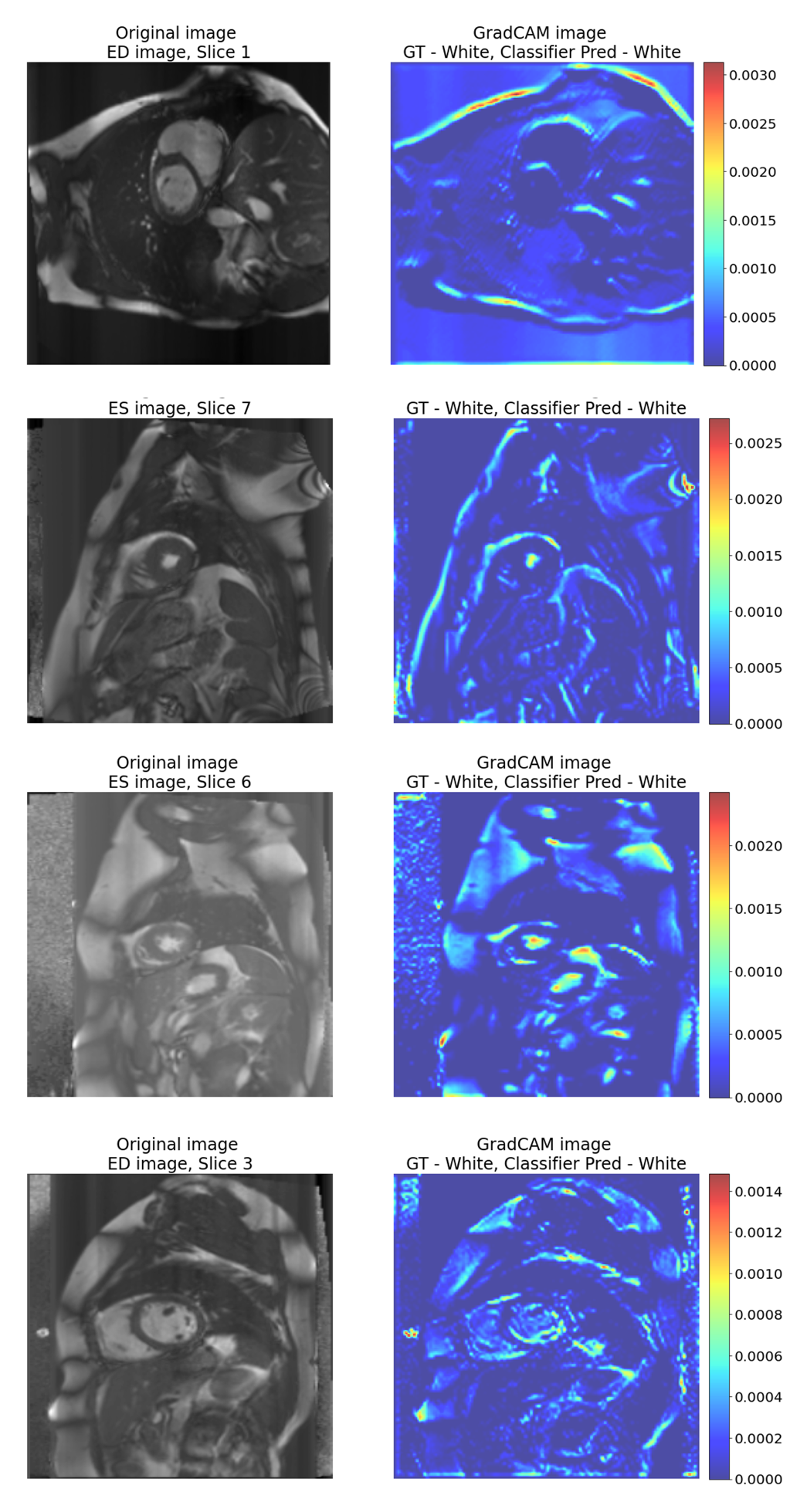


Supplementary Figure 3: Examples of normalised CMR images and GradCAM heatmaps for Im-Im-Im dataset for the White subjects in race classification experiments


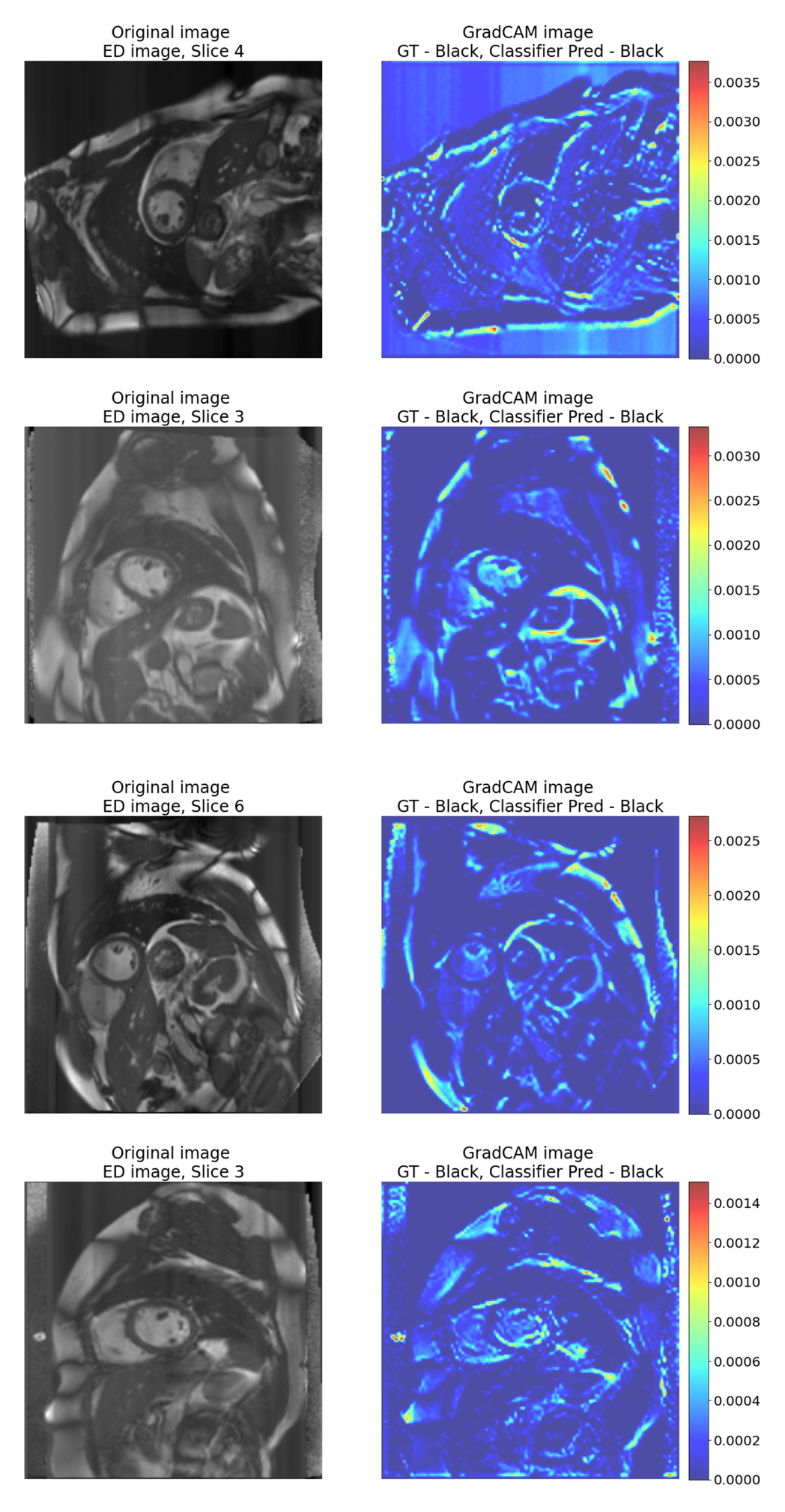


Supplementary Figure 4: Examples of normalised CMR images and GradCAM heatmaps for Im-Im-Im dataset for the Black subjects in race classification experiments

Supplementary Figure 5: Comparison of the prediction of right ventricular ejection fraction (RVEF), right ventricular end systolic volume (RVESV), left ventricular ejection fraction (LVEF) and left ventricular end systolic volume for the nnU-Net model using original images (left column) and cropped images (right column). Statistical significance was tested using a Mann-Whitney U test and is denoted by **** (p ≤ 0.0001), *** (0.001 < p ≤ 0.0001), ** (0.01 < p ≤ 0.001), * (0.01 < p ≤ 0.05), ns (0.05 ≤ p).

1. We repeated the classification and segmentation experiments using subjects balanced by age, BMI and sex (male and female). We matched the subjects in each group to subjects whose age and BMI were +/- 1 year and +/- 1 BMI point (BMI was rounded to the nearest whole number) (R1.5).

We repeated the classification experiment using the same setup as described previously and found an accuracy of 0.554, sensitivity of 0.588 and specificity of 0.519. We also repeated the segmentation experiments using the same setup as described previously. The results can be seen in Supplementary Figure 6. (R1.5)


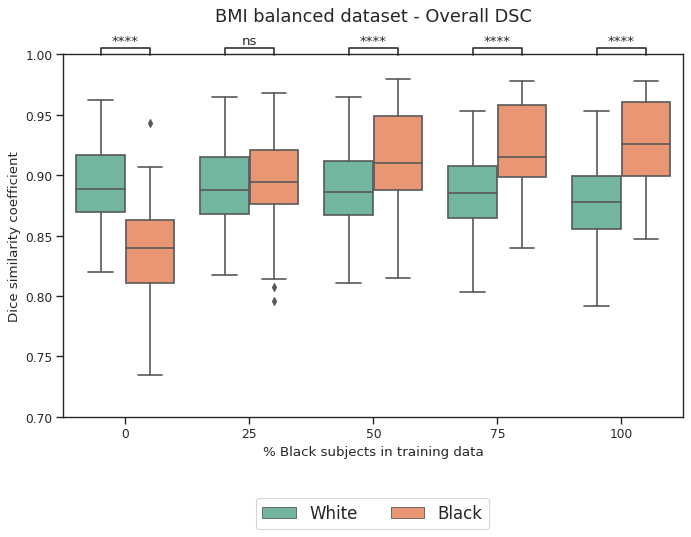


Supplementary Figure 6: Overall Dice similarity coefficient (DSC) for segmentation experiment using original CMR images. The subjects are controlled by age and BMI. Statistical significance was found using a Mann-Whitney U test and is denoted by **** (p ≤ 0.0001), *** (0.001 < p ≤ 0.0001), ** (0.01 < p ≤ 0.001), * (0.01 < p ≤ 0.05), ns (0.05 ≤ p). (R1.5)

1. We control for the MRI year of the subjects by matching the subjects’ age and MRI year to +/- 1 year.


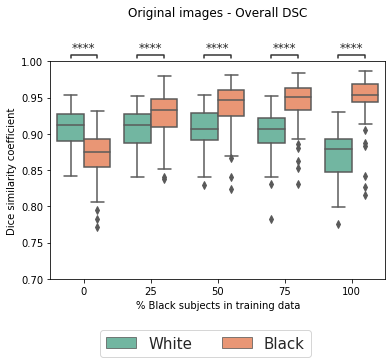


Supplementary Figure 7: Overall Dice similarity coefficient (DSC) for segmentation experiment using original CMR images. The subjects are controlled by age and MRI year. Statistical significance was found using a Mann-Whitney U test and is denoted by **** (p ≤ 0.0001), *** (0.001 < p ≤ 0.0001), ** (0.01 < p ≤ 0.001), * (0.01 < p ≤ 0.05), ns (0.05 ≤ p).
